# Do adults in contact with Australia's public sector mental health services get better?

**DOI:** 10.1186/1743-8462-3-9

**Published:** 2006-08-30

**Authors:** Philip Burgess, Jane Pirkis, Tim Coombs

**Affiliations:** 1 School of Population Health, The University of Queensland, Brisbane, Australia; 2 School of Population Health, The University of Melbourne, Melbourne, Australia; 3 New South Wales Institute of Psychiatry, Sydney, Australia

## Abstract

This paper describes the outcomes of episodes of care for adults in public sector mental health services across Australia, with a view to informing the debate on service quality. Health of the Nation Outcome Scales (HoNOS) change scores and effect sizes were calculated for 14,659 acute inpatient episodes and 23,692 community episodes. The results showed that people in contact with public sector mental health services generally do get better, although the magnitude of improvement depends on the setting and episode type. This confirmatory finding is particularly positive, given current community concerns about the quality and effectiveness of mental health services.

## Background

In Australia, as in other countries, there has been increasing concern about how well mental health services serve the community. Australia's National Mental Health Strategy has provided a strong policy platform for mental health service reform over the last decade or more [1], emphasising co-ordination between acute inpatient care and community services such that people can move between them according to their level of need. However, there is a view that this has not translated into optimal service delivery 'on the ground'. Some view this as a problem with the implementation of the Strategy [2], whereas others argue that the implementation is moving in the right direction, but that change may not have occurred extensively or quickly enough [3].

Various sources of evidence have been cited in the debate over the nature and quality of Australia's mental health services, and their capacity to meet consumers' needs. Key among these are several reports on the direct experiences of consumers and carers, which draw on the findings of large-scale consultations [4-6]. Also crucial are a series of National Mental Health Reports which have monitored the progress of the National Mental Health Strategy by providing data on changing patterns of mental health expenditure and service provision [7]. In addition, there have been two evaluations of the plans that operationalise National Mental Health Strategy, each of which relied on a combination of quantitative data (taken primarily from the National Mental Health Reports), and qualitative data (derived from consultations with key informants and commentary from international experts) [8,9].

What has been missing to date is any systematic evidence regarding whether people in receipt of mental health care improve. Consumer-level outcome data that can inform this question are necessary, and are now being collected by public sector inpatient and community mental health services in all Australian states/territories, and being routinely submitted by each state/territory to the Australian Government. Australia is an international leader in this regard – the United States, the United Kingdom and several continental European countries have also begun to routinely collect outcome data, but have not done so on such a comprehensive scale [10-12].

Collectively, the Australian outcomes data are known as the Mental Health National Outcomes and Casemix Collection (MH-NOCC). For adults, two clinician-rated outcome measures are used in each state/territory, along with one of three consumer-rated measures (see Table 1 for details). The Australian Mental Health Outcomes and Classification Network (AMHOCN) has been charged with the task of aggregating, analysing and reporting on these data [13]. The current paper describes the change in problem severity for adults in public sector acute inpatient and community mental health services across Australia, with a view to informing the debate on the quality of these services. The paper draws on a comprehensive report which is available on the MH-NOCC website, or from the authors on request [14,15].

**Table 1:** Outcome measures used for adults in Australian public sector mental health services

	**Rater**	**Overarching constructs**
**Health of the Nation Outcome Scales (HoNOS)**	Clinician	Mental health and social functioning
**Life Skills Profile 16 (LSP-16)**	Clinician	Disability
**Mental Health Inventory (MHI)**	Consumer	Psychological distress and wellbeing
**Behaviour and Symptom Identification Scale 32 (BASIS-32^®^)**	Consumer	Symptom and problem difficulty
**Kessler 10+ (K-10+)**	Consumer	Non-specific psychological distress

## Method

Under the MH-NOCC protocol, individual providers are responsible for administering the clinician-rated outcome measures (and offering consumers the consumer-rated measures) during given 'episodes of care', at particular 'collection occasions'. An episode is defined as '... a more or less continuous period of contact between a consumer and a mental health service organisation that occurs within the one mental health service setting [e.g., inpatient or community]' [16]. Collection occasions occur at set points and for different reasons: admission (new referral; transfer from other setting; other), at review (91 days; other), and at discharge (no further care; change of setting; death; other).

MH-NOCC data were available for the period July 2000 to April 2005. Data were provided at the collection occasion level, and then 'cleaned' and aggregated to 'episodes' by the AMHOCN study team. All episodes comprised more than one collection occasion, but it should be noted that not all episodes were bounded by admission and discharge collection occasions. The first collection occasion for those in ongoing treatment at the point of implementation of the national collection was a review, rather than an admission; and the last collection occasion for those in ongoing treatment when the MH-NOCC data were submitted was a review, rather than a discharge. In other words, certain valid sequences bounded episodes, creating the following episode sub-types: admission to review; admission to discharge; review to review; review to discharge.

The current paper focuses on one outcome measure only, the clinician-rated Health of the Nation Outcome Scales (HoNOS), developed by Wing and colleagues in the United Kingdom [17]. The HoNOS is a general measure of problem severity, and comprises 12 items that collectively cover the sorts of problems that may be experienced by people with a mental illness – namely, (1) Overactive, aggressive, disruptive or agitated behaviour; (2) Non-accidental self-injury; (3) Problem drinking or drug taking; (4) Cognitive problems; (5) Physical illness or disability problems; (6) Problems associated with hallucinations and delusions; (7) Problems with depressed mood; (8) Other mental and behavioural problems; (9) Problems with relationships; (10) Problems with activities of daily living; (11) Problems with living conditions; and (12) Problems with occupation and activities [17]. Psychometric studies have generally shown the HoNOS to have adequate to good validity, reliability, sensitivity to change and utility [18].

Each HoNOS item is rated from 0 (no problem) to 4 (very severe problem), resulting in individual item scores, subscale scores and a total score. HoNOS measures were included in the current analysis if at least 10 of the 12 items had valid clinical ratings. Missing items were treated as contributing 0 to the relevant subscale score, and to the total score.

HoNOS change scores and effect sizes were calculated for valid episode of care. The change score was defined as the mean difference between the 'start' and 'end' scores: a positive change score indicated a reduction in clinical severity (i.e., improvement) and a negative change score indicated an increase in clinical severity (i.e., deterioration). The effect size quantified the difference in change scores within episodes (i.e., was calculated from a repeated measures design perspective) [19], with an effect size of 0.00 indicating no change, an effect size of 0.20 considered 'small' and indicating negligible clinical importance, an effect size of 0.50 regarded as 'medium', indicating moderate clinical importance, and an effect size of 0.80 viewed as 'large', indicating critical clinical importance [20].

## Results

In total, data were available from 101,820 collection occasions from acute inpatient settings, and 183,071 from community settings. In the former setting, these collection occasions aggregated to 21,911 valid episodes, 14,659 (66.9%) of which had sufficiently complete HoNOS ratings at the first and last collection occasions. In the latter, they aggregated to 36,803 valid episodes, 23,692 (64.4%) with eligible HoNOS ratings.

Table 2 shows the HoNOS change scores for acute inpatient episodes, by episode sub-type. For episodes bounded by admission and discharge, which constitute the majority of acute inpatient episodes, the change score and effect size statistic provide evidence of improvement of 'critical clinical importance' (mean change score = 7.3; effect size = 1.00, 95%CI = 0.87–1.12). For episodes bounded by admission and review, the change score and effect size statistic indicate more modest, but still 'moderately clinically important' improvement (mean change score = 3.1; effect size = 0.43, 95%CI = 0.14–0.72). The same is true for episodes bounded by review and discharge (mean change score = 3.3; effect size = 0.48, 95%CI = 0.10–0.86). For episodes bounded by two reviews, the change score and effect size statistic indicate virtually no shift (mean change score = 0.4; effect size = 0.07, 95%CI = 0.05–0.10).

**Table 2:** HoNOS change scores for available episodes of acute inpatient care, Australia, July 2001 to April 2005

		**Start score**		**End score**		**Change score**		***Effect size statistics***		
	**VE**	**M**	**SD**	**M**	**SD**	**M**	**SD**	***d***	**LCI**	**UCI**
***Any Admission > Any Review***	***425***	***15.3***	***7.4***	***12.2***	***6.6***	***3.1***	***7.2***	***0.43***	***0.14***	***0.72***
New referral > 91-day review	99	14.7	8.6	11.8	6.7	2.9	6.8	0.42	-0.15	0.99
New referral > Other review	114	15.8	7.1	12.1	6.8	3.7	7.5	0.49	-0.18	1.16
From other setting > 91-day review	91	14.6	7.1	13.5	6.5	1.1	7.4	0.14	-0.08	0.36
From other setting > Other review	67	14.6	7.0	11.9	6.9	2.8	6.0	0.45	-0.20	1.11
Other > 91-day review	22	15.9	7.4	10.9	4.8	5.0	7.8	0.61	-1.49	2.72
Other > Other review	32	18.2	5.9	11.4	6.4	6.8	6.5	1.01	-1.32	3.34

***Any Admission > Any Discharge***	***13104***	***14.3***	***6.6***	***7.0***	***5.7***	***7.3***	***7.3***	***1.00***	***0.87***	***1.12***
New referral > No further care	1902	14.1	6.6	6.7	5.6	7.4	7.6	0.97	0.65	1.30
New referral > Change of setting	5840	14.4	6.7	7.3	6.1	7.1	7.5	0.94	0.76	1.12
New referral > Death	10	22.6	10.2	17.3	8.6	5.3	11.5	0.42	-2.98	3.82
New referral > Other	539	14.7	6.5	7.2	5.0	7.4	6.9	1.07	0.45	1.69
From other setting > No further care	519	14.2	6.7	6.9	5.8	7.2	7.1	1.01	0.40	1.63
From other setting > Change of setting	3229	13.9	6.4	6.4	5.1	7.5	7.0	1.07	0.81	1.32
From other setting > Death	4	17.0	13.0	14.8	8.9	2.3	5.6	0.29	-2.47	3.05
From other setting > Other	186	15.7	6.1	8.1	5.8	7.6	6.5	1.16	0.08	2.24
Other > No further care	101	13.8	6.4	6.6	5.0	7.1	6.6	1.07	-0.30	2.45
Other > Change of setting	387	14.9	6.9	7.3	5.3	7.5	7.1	1.05	0.31	1.80
Other > Death	0	-	-	-	-	-	-	-	-	-
Other > Other	387	15.8	6.2	8.5	4.8	7.3	6.3	1.16	0.44	1.88

***Any Review > Any Review***	***847***	***12.0***	***6.8***	***11.6***	***6.8***	***0.4***	***5.6***	***0.07***	***0.05***	***0.10***
91-day review > 91-day review	672	12.3	6.6	11.8	6.6	0.4	5.5	0.08	0.05	0.11
91-day review > Other	25	13.6	8.5	13.2	6.8	0.4	6.1	0.07	-0.11	0.25
Other > 91-day review	74	13.4	7.8	13.4	7.5	0.1	6.9	0.01	-0.02	0.03
Other > Other	76	8.3	5.5	7.8	6.9	0.5	5.3	0.10	-0.02	0.22

***Any Review > Any Discharge***	***283***	***12.1***	***6.7***	***8.8***	***6.6***	***3.3***	***6.9***	***0.48***	***0.10***	***0.86***
91-day review > No Further Care	48	9.3	5.0	6.8	5.3	2.5	4.9	0.51	-0.20	1.22
91-day review > Change of Setting	86	12.0	5.9	9.3	7.5	2.7	6.6	0.41	-0.16	0.98
91-day review > Death	2	14.5	4.9	11.0	9.9	3.5	4.9	-	-	-
91-day review > Discharge Other	4	15.5	4.5	12.3	5.8	3.3	1.9	1.25	-2.40	4.89
Other > No Further Care	21	13.0	7.5	10.2	7.2	2.8	8.8	0.30	-0.90	1.50
Other > Change of Setting	113	12.9	7.5	8.6	6.0	4.3	7.4	0.57	-0.21	1.36
Other > Death	2	11.0	12.7	21.5	0.7	-10.5	12.0	-	-	-
Other > Discharge Other	7	15.1	6.0	9.3	6.5	5.9	5.3	0.97	-3.59	5.52

*Explanatory Notes *

VE Valid Episodes of Care

SD Standard Deviation

N Valid Observations

*d *Unbiased Effect Size Estimator

- No Valid Observations

LCI Lower 95% Confidence Interval

M Mean

UCI Upper 95% Confidence Interval

Table 3 shows the HoNOS change scores for community episodes, by episode sub-type. For episodes bounded by admission and discharge, the change score and effect size statistic point to improvement of 'moderate clinical importance' (mean change score = 3.4; effect size = 0.52, 95%CI = 0.45–0.60). For episodes bounded by admission and review, the change score and effect size statistic indicate lesser improvement of 'small to moderate clinical importance' (mean change score = 1.8; effect size = 0.30, 95%CI = 0.25–0.35). This is also the case for episodes bounded by review and discharge (mean change score = 1.6; effect size = 0.28, 95%CI = 0.23–0.34). For episodes bounded by two reviews, the change score and effect size statistics indicate negligible improvement (mean change score = 0.7; effect size = 0.13, 95%CI = 0.12–0.15).

**Table 3:** HoNOS change scores for available episodes of community care, Australia, July 2001 to April 2005

		**Start score**		**End score**		**Change score**		**Effect size statistics**		
	**VE**	**M**	**SD**	**M**	**SD**	**M**	**SD**	***d***	**LCI**	**UCI**
***Any Admission > Any Review***	***4440***	***11.0***	***6.3***	***9.2***	***6.0***	***1.8***	***6.1***	***0.30***	***0.25***	***0.35***
New referral > 91-day review	2247	11.2	6.0	8.8	5.8	2.5	5.7	0.44	0.33	0.54
New referral > Other review	538	12.7	6.5	10.8	6.7	1.9	6.7	0.28	0.12	0.44
From other setting > 91-day review	1107	9.7	6.2	8.9	6.1	0.7	6.5	0.11	0.07	0.16
From other setting > Other review	109	11.7	7.2	10.4	6.3	1.2	6.1	0.20	-0.03	0.42
Other > 91-day review	399	10.6	6.4	9.3	5.9	1.3	5.6	0.22	0.10	0.35
Other > Other review	40	11.2	6.2	10.3	5.6	0.9	6.0	0.15	-0.13	0.43

***Any Admission > Any Discharge***	***8235***	***11.3***	***6.1***	***7.9***	***6.5***	***3.4***	***6.5***	***0.52***	***0.45***	***0.60***
New referral > No further care	4039	11.3	5.8	6.3	5.3	5.0	5.4	0.93	0.78	1.08
New referral > Change of setting	1485	12.4	6.3	10.6	7.1	1.7	6.2	0.28	0.19	0.37
New referral > Death	9	16.0	7.1	14.6	9.8	1.4	6.8	0.19	-0.80	1.18
New referral > Other	597	10.8	5.9	6.8	5.7	4.0	4.9	0.81	0.50	1.12
From other setting > No further care	970	10.8	6.0	6.9	5.5	3.8	6.2	0.62	0.38	0.86
From other setting > Change of setting	664	10.1	6.6	14.2	7.4	-4.1	8.7	-0.47	-0.77	-0.16
From other setting > Death	7	11.0	8.4	16.0	7.4	-5.0	6.4	-0.68	-4.59	3.23
From other setting > Other	31	12.9	6.5	8.7	6.4	4.2	5.4	0.75	-0.71	2.22
Other > No further care	131	10.9	6.4	6.0	5.3	4.9	5.3	0.92	0.09	1.75
Other > Change of setting	85	13.5	7.2	13.4	8.1	0.1	6.6	0.02	-0.01	0.06
Other > Death	2	17.5	9.2	20.0	28.3	-2.5	19.1	-	-	-
Other > Other	215	11.1	5.8	7.3	5.8	3.8	4.9	0.78	0.28	1.28

***Any Review > Any Review***	***8073***	***9.5***	***6.1***	***8.8***	***5.9***	***0.7***	***5.2***	***0.13***	***0.12***	***0.15***
91-day review > 91-day review	6323	9.3	6.0	8.6	5.9	0.6	5.0	0.13	0.11	0.14
91-day review > Other	405	10.1	6.4	9.8	5.9	0.3	6.4	0.05	0.02	0.08
Other > 91-day review	798	10.2	6.0	9.0	5.6	1.2	5.3	0.23	0.15	0.32
Other > Other	547	11.1	6.3	10.4	6.5	0.7	5.4	0.14	0.08	0.20

***Any Review > Any Discharge***	***2944***	***8.9***	***6.0***	***7.3***	***6.5***	***1.6***	***5.6***	***0.28***	***0.23***	***0.34***
91-day review > No Further Care	1465	8.1	5.5	5.4	5.0	2.6	4.5	0.58	0.45	0.72
91-day review > Change of Setting	733	10.0	6.5	11.1	7.5	-1.1	6.5	-0.17	-0.24	-0.09
91-day review > Death	18	11.1	5.7	12.7	7.7	-1.7	5.1	-0.31	-1.08	0.46
91-day review > Discharge Other	167	8.6	6.0	7.1	6.0	1.5	5.2	0.29	0.06	0.52
Other > No Further Care	334	9.3	5.9	5.9	5.0	3.5	5.2	0.66	0.29	1.02
Other > Change of Setting	163	11.2	6.5	10.7	7.4	0.5	5.8	0.08	0.01	0.15
Other > Death	2	8.5	2.1	3.5	3.5	5.0	1.4	-	-	-
Other > Discharge Other	62	9.1	6.7	6.9	6.2	2.1	6.6	0.32	-0.21	0.85

*Explanatory Notes *

VE Valid Episodes of Care

SD Standard Deviation

N Valid Observations

*d *Unbiased Effect Size Estimator

- No Valid Observations

LCI Lower 95% Confidence Interval

M Mean

UCI Upper 95% Confidence Interval

Tables 2 and 3 both provide a further breakdown of the change scores within each of the four episode sub-types described above, presenting results at the level of reason for collection occasion within each. There is some variability within each episode sub-type. So, for example, for community episodes bounded by admission and discharge, the improvement is universally greater in episodes in which the reason for discharge is no further care or 'other' than in episodes where the discharge is to a changed setting or death (where in fact there is sometimes deterioration).

Also of note in Tables 2 and 3 are the mean start and end scores for each episode sub-type, from which the change scores are derived. They indicate that, as would be expected, the typical level of severity of symptoms is higher for people admitted to acute inpatient episodes than those admitted to community episodes. There is less difference between severity levels at discharge from the two settings. Taken together, these findings indicate that there is greater scope for improvement in the acute inpatient setting, but that the overall outcome may be approximately the same.

## Discussion

The findings suggest that, on the whole, people in contact with Australia's public sector mental health services do get better. The magnitude of improvement is greater in acute inpatient episodes than in community episodes. This makes sense, given that people who are admitted to the former setting would typically experience greater problem severity than those admitted to the latter, and would therefore have greater room for improvement. In both acute inpatient and community settings, the extent of improvement is greater in episodes that begin with admission and end with discharge, than it is in episodes that are punctuated at one end or the other by a review. Again, this finding has good face validity, in the sense that improvement would be expected to occur on a gradient. Within settings, the patterns of improvement are also intuitively sensible.

Some caution should be exercised in interpreting the above findings, since they cannot be interpreted as causal. To demonstrate that mental health services were making people better would require a different study design, and would require evidence that those who were not in contact with mental health services did not show similar degrees of improvement.

In addition, the study had several limitations related to data volume and quality. Firstly, although all Australian states/territories are now submitting MH-NOCC data, the progress with which individual jurisdictions have implemented routine outcome measurement has not been uniform, so the majority (almost three quarters) of collection occasions were recorded in 2003–04 and 2004–05. Secondly, there was no way of knowing the level of compliance with administering the HoNOS, since no denominator data were available on the total number of consumers under care. Thirdly, some collection occasions were 'dropped' at the point of converting them to episodes (because they did not constitute valid sequences) and some episodes were not included in the analysis (because they did not include the requisite valid HoNOS ratings), which may have introduced systematic biases into the results if particular types of consumers were more likely to be the focus of these collection occasions or episodes. Fourthly, the reliability and validity of the HoNOS ratings are arguably open to question, although it should be noted that clinicians across the country have been trained in the instrument's use, and there are no financial incentives for 'gaming' [13].

These limitations aside, the findings address an evidence gap in a way that can inform the debate on how well Australia's public sector mental health services are serving the community. Until now, no comprehensive data have been available on consumer outcomes; the current study provides such data, and suggests that, in general, these outcomes are positive. This is particularly impressive, given that these services primarily provide treatment for people with psychotic and affective disorders [21], which can be chronic and disabling. Of course, the aggregate nature of the data means that the results are reported as averages, so there will be some consumers who do not experience improvements and may even deteriorate during contact with mental health services. Equally, the data do not address the question of unmet need – and in particular the availability of services in times of crisis – which is of concern to many [4-6]. In other words, the data should not be over-interpreted since they represent only one piece of the jigsaw, but they do suggest that, in the main, people in contact with Australia's public sector mental health services get better.

The study demonstrates the value of routine outcome measurement, in terms of informing questions of service quality and effectiveness. Routine outcome measurement was introduced in Australia under the National Mental Health Strategy, in recognition of the fact that the quality and effectiveness of services can only be achieved through the development of sound information to support service planning and delivery. In mental health circles, Australia is considered a world leader in this regard [22]. Other areas of health could learn from the experiences of the mental health sector in implementing routine outcome measurement and using the data to address key policy and planning questions, both within Australia and overseas [23].

The current study provides a global picture of adult consumers' improvement in public sector acute inpatient and community mental health services, as measured by the HoNOS. The challenge for mental health services is now to use this information to inform service and program development to support high quality care. There is also a need for further research endeavours, and future work by the AMHOCN study team will pursue more detailed analysis of the above data (e.g., stratifying by factors such as age, sex and diagnostic group, and controlling for factors such as the initial HoNOS profile), consider additional consumer groups (children and adolescents; older people) and additional settings (community residential services), and explore outcomes assessed by different instruments, both clinician-rated and consumer-rated.

To conclude, the current study suggests that adults in contact with public sector acute inpatient and community mental health services experience a reduction in problem severity during the course of a typical episode of care. While one would hope that this would be the case, it has not been demonstrated in any systematic way before. This confirmatory finding is particularly positive, given current community concerns about the quality and effectiveness of mental health services.

## Declaration of competing interests

The author(s) declare that they have no competing interests.

## Authors' contributions

PB, JP and TC conceptualised the idea for the paper, PB undertook the analyses, and PB, JP and TC all prepared the manuscript. All authors read and approved the final manuscript.
